# Remote ischemic preconditioning improves cognitive control in healthy adults: Evidence from an event-related potential study

**DOI:** 10.3389/fnins.2022.936975

**Published:** 2022-08-09

**Authors:** Yaling Li, Pei Huang, Jun Huang, Zhifeng Zhong, Simin Zhou, Huaping Dong, Jiaxin Xie, Yu Wu, Peng Li

**Affiliations:** ^1^Department of High Altitude Operational Medicine, College of High Altitude Military Medicine, Army Medical University, Chongqing, China; ^2^Key Laboratory of Extreme Environmental Medicine, Ministry of Education of China, Army Medical University, Chongqing, China; ^3^Key Laboratory of High Altitude Medicine, Army Medical University, Chongqing, China; ^4^Chongqing Key Laboratory of Psychological Diagnosis and Education Technology for Children With Special Needs, College of Education Science, Chongqing Normal University, Chongqing, China

**Keywords:** remote ischemic preconditioning, cognitive control, classic Stroop task, ERPs, N450, SP

## Abstract

It is suggested that remote ischemic preconditioning (RIPC) may be a promising treatment for improving healthy adults’ cognitive control. However, direct empirical evidence was absent. Therefore, this study aims to provide evidence for the impact of RIPC on cognitive control. Sixty healthy young male volunteers were recruited, and 30 of them received 1-week RIPC treatment (RIPC group), while the rest did not receive RIPC (control group). Their cognitive control before and after RIPC treatment was evaluated using the classic Stroop task, and the scalp electricity activity was recorded by event-related potentials (ERPs). The behavioral results showed a conventional Stroop interference effect of both reaction times (RTs) and the accuracy rate (ACC), but the Stroop interference effect of RTs significantly decreased in the posttest compared to the pretest. Furthermore, at the electrophysiological level, ERP data showed that N450 and SP for incongruent trials were larger than that for congruent trials. Importantly, the SP differential amplitude increased after RIPC treatment, whereas there was no significant change in the control group. These results implied that RIPC treatment could improve cognitive control, especially conflict resolving in the Stroop task.

## Introduction

One prominent advantage humans have over animals is that our action is intentional and not entirely driven by the environmental stimulus. The process underlying such adaptability was cognitive control (also called executive function) (Botvinick et al., 2001), which was defined as the ability to coordinate thought and behavior in accord with internally represented intentions and plans (Miller and Cohen, 2001). Substantial studies suggested that the prefrontal cortex (PFC) played a crucial role in this cognitive function (Miller and Cohen, 2001; Koechlin et al., 2003). Many neuroimaging studies confirmed that when participants accomplish tasks requiring cognitive control, their PFC was significantly activated (Braver et al., 2003; Koechlin et al., 2003; Braver, 2012). Meanwhile, results from lesion studies showed that individuals with PFC focal damage had a poor performance in tasks involving cognitive control (Miller, 2000; Gläscher et al., 2012).

Cognitive control subserves higher cognition processes, such as attention, thinking, planning, and reasoning in our daily life (Koechlin et al., 2003). Indeed, individuals with mood or behavior disorders have been proved to be deficient in cognitive control. For example, individuals with anxiety and depression disorders were more likely to be interfered with by unrelated threatening stimuli compared to non-threatening ones (Pishyar et al., 2004; Hofmann, 2007; Staugaard, 2010), which was associated with impaired cognitive control (Hallion et al., 2017). Additionally, Stewart et al. (2017) proposed that the increase in suicide behaviors among adolescents might also be related to general deficits in cognitive control, which generally did not have enough time to get mature (Luna, 2009). Therefore, a variety of psychology and clinical research was pursued to improve individuals’ cognitive control. Currently, there are a variety of effective methods. For example, well-designed video game training could contribute to cognitive control in older adults (Anguera et al., 2013), and non-invasive current stimulation was also reported (Hsu et al., 2011; Jacobson et al., 2012). Interestingly, the musical experience also could enhance cognitive control (Slevc et al., 2016; Sharma et al., 2019), probably because inhibitory control and attention shift play crucial roles in playing music (Slevc et al., 2016). However, these methods might also have some deficiencies. For example, video game training might lead to addiction, especially for adolescents with immature cognitive control, and the current stimulation might not be accepted by some populations owing to its unsafety (Antal et al., 2017). Musical training is demanding, and musical instrument playing is highly professional, thus it might take a long time for individuals to get benefit from this method.

Interestingly, Sugimoto et al. (2021) recently reported that the blood flow restriction, similar to ischemic preconditioning (IPC), could improve healthy adults’ cognitive control when combined with a walking train, thus proposing that the clinical IPC may be a potential treatment for improving cognitive control of healthy adults without exercise. IPC is a systemic strategy characterized by a brief episode of ischemia that renders the target organs more resistant to subsequent longer ischemic events (Murry et al., 1986). Its direct stress on target organs, such as the brain itself, however, would be more challenging and less practical than to other organs, and also lead to mechanical trauma to major vascular structures, which has limited its clinical application (Tapuria et al., 2008). As the derivative of IPC, remote ischemic preconditioning (RIPC) did not stress to target tissue directly but protected it through several transient and non-lethal limb ischemia of distant organs (Jensen et al., 2011; Moskowitz and Waeber, 2011; Zhao et al., 2017). In comparison to IPC, RIPC is inexpensive, safe, and non-invasive (Moskowitz and Waeber, 2011), which has been widely used in clinical brain injury treatment and cognitive function improvement. RIPC could protect the brain against injury caused by various diseases (Jensen et al., 2011; Zhao et al., 2018), for instance, Zhao et al. (2018) reported that RIPC could improve cerebral circulation in patients with symptomatic intracranial arterial stenosis. Additionally, RIPC also plays a critical role in protecting cognitive function after brain injury (Hudetz et al., 2015; He et al., 2017; Wang et al., 2017), for example, Wang et al. (2017) showed that RIPC alleviated cognitive function impairment in patients with the cerebral small-vessel disease.

However, to data, there is no direct evidence of the improvement of healthy adults’ cognitive control through RIPC treatment, because in these existing studies, RIPC was applied to improve the cognitive control of these individuals who undergoing surgery or in special circumstances. Furthermore, these studies are very scant and their results are inconsistent (Meybohm et al., 2013, 2018; Hudetz et al., 2015; Li et al., 2020; Sugimoto et al., 2021). For instance, in the investigations by Meybohm et al. (2013, 2018), the patients who underwent a wide range of cardiac surgery accomplished a Stroop test (a test demanding cognitive control) before and after receiving the RIPC intervention (induced by four cycles of upper-limb ischemia, 5 min), aim to examine the effect of RIPC on reducing postoperative neurocognitive dysfunction, as a result, they failed to demonstrate the efficacy of a RIPC protocol on cognitive control. Hudetz et al. (2015) reported that the cognitive control tested by the Stroop task before and after surgery were not changed in individuals who both accepted and rejected the RIPC treatment (four cycles of brief upper extremity ischemia, 5 min) but found that the RIPC treatment can prevent deterioration of other short-term cognitive abilities, such as non-verbal and verbal memory. Li et al. (2020) found that the alertness but not the executive function of attention in the attentional network test (ANT) was changed by RIPC (5 min bilateral upper limbs ischemia and 5 min reperfusion for five cycles, twice a day, for 7 days) in adults unacclimatized to high altitude. In summary, except for the study by Sugimoto et al. (2021), the above four research did not observe the protective effect of RIPC on cognitive control. One of the most possible reasons was that any slight effect of RIPC was masked by the negative impact of the surgery or hypoxia itself in these studies (Meybohm et al., 2018).

The present study aimed to demonstrate the possible impact of RIPC on cognitive control, if any, which may provide an alternative treatment for improving cognitive control. To prevent the effect of RIPC that was canceled out by surgery or other factors, healthy adults were recruited and their cognitive control was measured in a normal environment. Furthermore, previous research did not find a positive effect of RIPC on cognitive control at the behavioral level (e.g., Meybohm et al., 2013, 2018; Li et al., 2020). One possible reason was that behavioral indicator, such as reaction times (RTs) and the accuracy rate (ACC) only a gross reflection of the cognitive process, which is insensitive to minor distinctions. Hence, in our study, besides behavioral data, the participants’ scalps’ electrical activity was also recorded via event-related potentials (ERPs). ERP reflected electrical activity locked to a specific task event or response and offered a real-time temporal resolution of neural processes for the task performance, which could be used to investigate the time course of cognitive processing (Hillyard and Anllo-Vento, 1998). Undoubtedly, ERP was a more sensitive indicator for the subtle distinction in the cognitive process than RTs and ACC.

The RIPC treatment scheme was based on previous schemes and combined with the reality of this study: four cycles of RIPC therapy were performed daily for 7 days with 5 min of bilateral upper limb ischemia (180 mm Hg) and 5 min of reperfusion (Li et al., 2020). This study adopted the classic Stroop task (Stroop, 1935) to assess participants’ cognitive control before and after receiving RIPC. In the Stroop task, participants were required to name the color of the word and ignore its meaning. Under the congruent condition, words have the same color and meanings (e.g., the “red” word printed with red ink). Under the incongruent condition, the word color and meanings were different (e.g., the “red” word printed with green ink), compared with the congruent conditions, the participant’s RTs would take longer and ACC would decrease to name the ink color under the incongruent conditions, known as the Stroop interference effect (MacLeod, 1991). The mechanism of the Stroop interference effect was proposed to reflect the competition between the control brain system and the automatic processing brain system (Cohen et al., 1990). Accessing the meaning of words was automatic while naming the color was controlled. Therefore, the meaning of the word was acquired preferentially than its color, leading to the conflict between the stimulus and response under the incongruent condition, so the conflict must be resolved at the cost of slowing their response (Cohen et al., 1990).

Two ERP components, N450 and sustained potential (SP) were reported in the Stroop task, which are the two markers of conflict processing (Liotti et al., 2000; West et al., 2005; Larson et al., 2009, 2014). N450 was a negative wave peaking at about 450 ms after the stimuli presentation over fronto-central electrodes (West et al., 2005). A wealth of studies showed that N450 elicited by incongruent trials was greater than that of congruent trials, reflecting the process of conflict detection (Szûcs and Soltész, 2010; Tillman and Wiens, 2011; Larson et al., 2014). ERPs source localization analysis implied that the N450 was mainly derived from the anterior cingulate cortex (ACC) (Van Veen and Carter, 2002; Hanslmayr et al., 2008). The SP was identified in the incongruent vs. congruent difference wave succeeding the N450 (Liotti et al., 2000; West and Alain, 2000; West, 2003; Lansbergen et al., 2007). SP is more positive for incongruent trials than for congruent trials or errors counterparts in the Stroop task, leading to the suggestion that the conflict SP reflects the process of conflict resolution (Liotti et al., 2000; West and Alain, 2000; West et al., 2005; Larson et al., 2009). SP was presumably generated from the dorsal lateral prefrontal cortex (dlPFC) (Lansbergen et al., 2007). In summary, these ERP findings indicated that conflict processing in the Stroop task includes conflict monitoring and resolution, represented by N450 and SP, respectively.

We hypothesize that participants’ cognitive control will be enhanced after 1 week of RIPC treatment (performed once a day for 7 consecutive days, 5 min ischemia with 180 mm Hg cuff pressure, followed by 5 min reperfusion). The greater the difference between incongruent and congruent conditions of N450 and SP, the better would be conflict monitoring and resolution ability (Badzakova-Trajkov et al., 2009; Larson et al., 2014). Therefore, we predict that the enhanced cognitive control would be reflected by a larger N450 or SP differential amplitude.

## Materials and methods

### Participants

**FIGURE 1:**
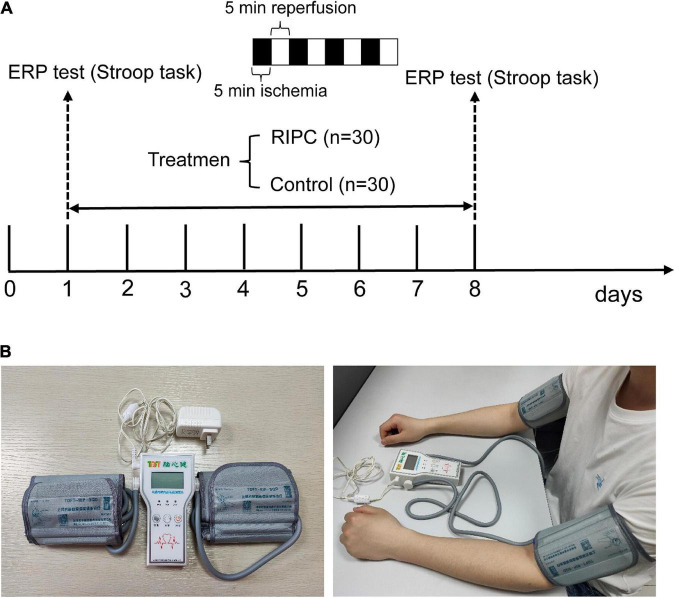
The schematic diagram of the whole procedure, RIPC device, and the usage diagram. **(A)** The Stroop task and event-related potentials (ERPs) were adopted to evaluate cognitive control ability before and after RIPC treatment. The RIPC group (*n* = 30) was assigned to accept 40 min per day for a total of 7 days of RIPC treatment, four cycles of 5 min ischemia (180 mmHg) and 5 min reperfusion were performed on the bilateral upper limb, and the control group (*n* = 30) were not accepted RIPC. **(B)** The RIPC device (left image) and how it is used (right image), the consent statement about the human image has been approved.

We used the G*power software to calculate the number of subjects needed in this study before the sample size was determined. The calculated parameters are as follows: effect size *f*: 0.25, α err prob: 0.05, power (1-β err prob): 0.8, number of groups: 2, and number of measurements: 4. The total sample size was calculated to be 24. Thus, a total of 60 healthy Chinese male participants were enrolled in this study. All participants were right-handed, as well as had normal or corrected vision. Additional inclusion criteria included no history of neurological or psychiatric disorders, no color weakness or color blindness, and could well tolerate RIPC. The participants were randomly divided into two groups: the RIPC group (*n* = 30) receiving RIPC and the control group (*n* = 30) without RIPC treatment. The schematic diagram of the whole procedure is shown in Figure 1A. Twelve participants were excluded due to excessive Electroencephalogram (EEG) artifacts or technical problems during EEG data preprocessing, leading to more than half of the ERP epochs being discarded. Finally, 20 (control group) and 28 (RIPC group) participants’ behavioral and electrophysiological data were further analyzed. There was no significant difference in the age (*p* = 0.41), weight (*p* = 0.13), height (*p* = 0.24), and Body Mass Index (BMI) between the RIPC [mean age = 20.50 years, standard deviation (SD) = 1.35; mean weight = 65.48 kg, SD = 9.33; mean height = 171.35 cm, SD = 6.49; mean BMI = 22.27 kg/m^2^, SD = 2.66] and control group (mean age = 20.85 years, SD = 1.53; mean weight = 62.35 kg, SD = 4.61; mean height = 169.55 cm, SD = 3.97; mean BMI = 21.71 kg/m^2^, SD = 1.75). The study was approved by the Medical Ethics Committee of Army Medical University. All ethical principles were stringently followed during the entire course of the study. Moreover, all participants signed a consent form before starting the experiment.

### Intervention

The RIPC treatment consisted of four cycles of bilateral upper limb ischemia for 5 min followed by reperfusion for 5 min, performed once a day for 1 week. The treatment was carried out using an automatic electric control device; the device and its usage are shown in Figure 1B. Limb ischemia was induced by inflating blood pressure cuffs to 180 mmHg. The Stroop task was performed before and after the RIPC treatment. In the event of discomfort or lack of tolerance, the participants could abort the RIPC process at any time. The control group underwent the same process, except without the RIPC treatment.

### Stimulus and procedure

**FIGURE 2:**
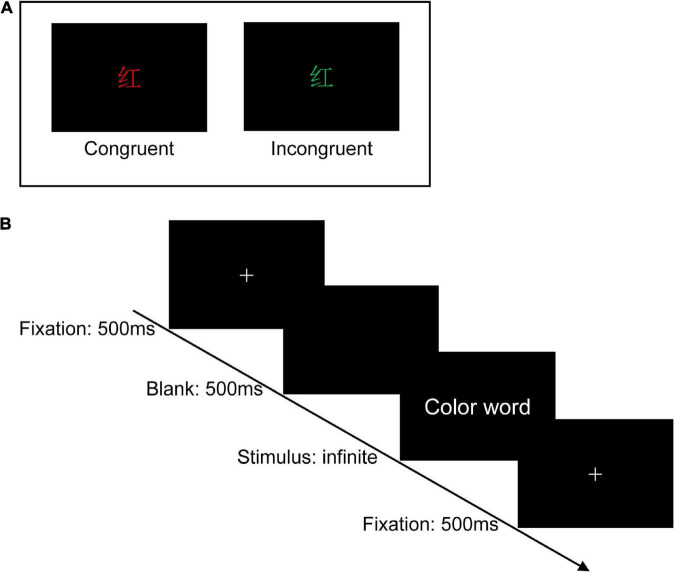
Experimental design. **(A)** Two examples of the Stroop task (Chinese character “
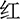
” means red). **(B)** Single-trial settings. During each trial, a white cross was first shown on the center of the black background for 500 ms, followed by a blank screen (500 ms), and then the color words appeared on the center of the black background, which was remained on the screen until participants pressed the specific key.

The experiment was conducted with the software E-Prime3.0. Participants were presented with the Chinese color words “
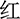
” (red), “
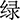
” (green), and “
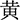
” (yellow) printed in different colors on the center of the computer screen. The stimulus under the congruent condition was color words printed in the congruent ink color [e.g., “
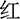
” (red) printed in red color], and the stimulus under the incongruent condition is the same Chinese character but printed with incongruent colors [i.e., “
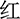
” (red) printed in yellow or green color], two examples of the Stroop task as shown in Figure 2A. The three colors (red, yellow, and green) were randomly mapped to three keys on the computer keyboard (j, k, and l). The participants were instructed to respond to the ink colors of the word and ignore its meaning by pressing one of three keys, and the responding keys were balanced between the participants.

In a dimly lit and sound-attenuated room with only the participant seated in, stimuli were presented on a 14-inch Lenovo laptop at a resolution of 1,920 × 1,080 pixels, with a refresh rate of 60 Hz. The distance between the participants and the computer monitor was 70 cm approximately. Before the formal experiment, the experimenter explained the overall process and details to the participants and required them to practice 20 trials to ensure they had fully understood the operation of the Stroop task. There are 180 trials in total in the formal Stroop task, 90 trials for the congruent condition (three kinds of stimulus, each was repeated 30 times) and 90 trials for the incongruent condition (six kinds of stimulus, each was repeated 15 times). The 180 trails were divided into three blocks and each contained 60 trials. Half of the trials were congruent and the other half were incongruent. The trials were mixed and presented in random order. During each trial, a white cross was first shown on the center of the black background for 500 ms, followed by a blank screen (500 ms), and then the color words appeared on the center of the black background, which remained on the screen until participants pressed the specific key. After accomplishing one block, each subject would have a short rest. The experiment procedure is shown in Figure 2B.

### EEG acquisition and preprocessing

The data were recorded using a BioSemi ActiveTwo system with a 64 Ag-AgCl Active-electrode array (BioSemi B.V., Amsterdam, Netherlands; for exact position coordinates).^1^ These coordinates were converted into the extended international 10–20 system, and electrode offsets were kept below 50 mV during recording, with a sampling frequency of 1,024 Hz.

EEG data analyses were performed in MATLAB (R2019b, The MathWorks, Inc., Natick, United States) with custom-made scripts supported by EEGLAB (Delorme and Makeig, 2004). Continuous EEG data were down-sampled to 500 Hz, re-referenced offline to the average of the activity recorded at the whole brain, and were then digitally band-pass filtered at 0.1–40 Hz, then the data were segmented to epochs from –1,000 to 2,000 ms around the target onset. The average value in the 200-ms time window preceding stimulus onset was used for baseline correction. First, all errors and extreme epochs (RTs exceeding ± 3 SD of mean) were excluded. Next, independent component analysis (ICA) was computed to isolate artifacts in the EEG signal using the logistic infomax ICA algorithm in the runica function of EEGLAB. Independent components representing eye blinks and movements, muscle artifacts, or other types of noise automatically identified by IClabel plugins in EEGLAB were removed from the data (Pion-Tonachini et al., 2019). Finally, the preprocessed EEG data were re-examined visually to ensure that significant artifacts were removed and at least 60 trials were available for each subject. The mean number of trials in each condition after excluding all artifacts as follows: congruent condition in pretest (Control vs. RIPC = 68 vs. 77), incongruent condition in pretest (Control vs. RIPC = 69 vs. 79), congruent condition in posttest (Control vs. RIPC = 69 vs. 80), and incongruent condition in posttest (Control vs. RIPC = 71 vs. 78).

### Data analysis

For the behavioral data, these trials of errors and RTs exceeding ± 3 SD of mean were first excluded. Next, a three-way repeated measure ANOVA was done for ACC and RTs, using the group (Control/RIPC) as a between-subject variable, time (Pretest/Posttest), and condition (Congruent/Incongruent) as the within-subjects variable. Then, we calculated the Stroop interference effect of ACC and RTs for each participant, which was defined as the difference between congruent and incongruent conditions separately (MacLeod, 1991). Subsequently, a 2 (group: Control/RIPC) × 2 (time: Pretest/Posttest) repeated measures ANOVA was done for the Stroop interference effect of ACC and RTs, using the group as a between-subject variable and time as a within-subjects variable.

For the ERP data, we combined our grand average ERP waveforms and topographical map with previous studies to select the measure time window and region of interest (ROI) for calculating the amplitude of each component. The ROI of the N450 component was selected as fronto-central including FCz, FC1, FC2, and Cz electrodes (Tillman and Wiens, 2011), and the mean amplitude of N450 between 400 and 600 ms was calculated by averaging these electrodes. For the SP component, the ROI was selected as the parietal region, where was reported record the maximal conflict SP amplitudes (Larson et al., 2009), including Pz, P1, P2, P3, and P4 electrodes, and SP were computed as mean amplitude in the time window of 600–1,000 ms after these electrodes were averaged. We performed a 2 (group: Control/RIPC) × 2 (time: Pretest/Posttest) × 2 (condition: Congruent/Incongruent) repeated measures ANOVA for each component, using the group as a between-subject variable and time and condition as the within-subjects variable.

All statistical analyses were performed using the SPSS, 28.0 version. A two-tailed significance level of 0.05 was used for all statistical tests and *p*-values were corrected for sphericity assumption violations using the Greenhouse-Geisser correction, and partial eta-squared (η_p_^2^) was calculated to describe effect sizes.

## Results

### Behavioral results

The raw data of ACC and RTs in each condition/group are presented in Table 1.

**TABLE 1:** The raw behavioral data in pretest and posttest of control and RIPC group.

Behavioral data	Group	Pretest (M ± SD)		Posttest (M ± SD)	
		Congruent	Incongruent	Congruent	Incongruent
**ACC (%)**	Control (*n* = 20)	97.17 ± 2.92	96.11 ± 3.41	97.61 ± 2.82	96.67 ± 3.28
	RIPC (*n* = 28)	97.90 ± 2.04	95.48 ± 4.18	98.02 ± 1.82	95.56 ± 4.11
**RTs (ms)**	Control (*n* = 20)	714.05 ± 154.05	839.11 ± 169.41	629.98 ± 97.97	727.22 ± 102.32
	RIPC (*n* = 28)	717.41 ± 164.12	888.51 ± 268.26	683.44 ± 142.93	800.80 ± 207.71

Values are presented as mean ± SD; n, number of participants; ACC, the accuracy rate; RTs, reaction times; Pretest, before treatment; Posttest, immediately after treatment.

**FIGURE 3:**
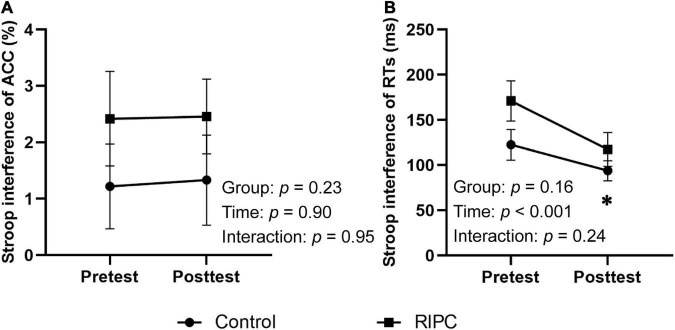
Behavioral results of two groups. **(A)** Stroop interference effect of ACC. **(B)** Stroop interference effect of RTs. Error bar represents the stand error; ACC, the accuracy rate; RTs, reaction times; Pretest, before treatment; Posttest, immediately after treatment. *Significant difference (*P* < 0.001) between time points.

For a three-way repeated measure ANOVA of ACC, only the main effect of congruency reached significance, *F*(1,46) = 13.96, *p* < 0.001, η_p_^2^ = 0.23, showing that participants’ ACC was higher for congruent trials than for incongruent trials. The main effect of time, *F*(1,46) = 0.38, *p* = 0.54, η_p_^2^ < 0.01, the two-way interactions of time by group [*F*(1,46) = 0.11, *p* = 0.74, η_p_^2^ < 0.01], condition by group [*F*(1,46) = 1.51, *p* = 0.23, η_p_^2^ = 0.03] and time by condition [*F*(1,46) = 0.02, *p* = 0.90, η_p_^2^ < 0.001], and the three-way interaction of group by time by condition [*F*(1,46) < 0.01, *p* = 0.96, η_p_^2^ < 0.001] were not significant. For the Stroop interference effect of ACC, the main effects of time [*F*(1,46) = 0.02, *p* = 0.90, η_p_^2^ < 0.001] and group [*F*(1,46) = 1.51, *p* = 0.23, η_p_^2^ = 0.03], the two-way interaction of time by group [*F*(1,46) < 0.01, *p* = 0.95, η_p_^2^ < 0.001] was not significant, as shown in Figure 3A.

For a three-way repeated measures ANOVA of RTs, main effects of both time [*F*(1,46) = 19.60, *p* < 0.001, η_p_^2^ = 0.30] and condition [*F*(1,46) = 102.59, *p* < 0.001, η_p_^2^ = 0.69] were discovered. For the time effect, participants responded faster to the ink color of words in the posttest (710.36 ms) than to the pretest (789.77 ms). Furthermore, for the condition effect, participants’ RTs for incongruent trials (813.91 ms) were longer than that for congruent trials (686.22 ms). There was also a two-way interaction of time and condition, *F*(1,46) = 15.11, *p* < 0.001, η_p_^2^ = 0.25, and further simple effects analysis showed participants responded more slowly to incongruent trials (pretest: 715.73 ms, posttest: 656.71 ms) than to congruent trials (pretest: 863.81 ms, posttest: 764.01 ms), but this difference was smaller in the posttest (107.30 ms) than that in the pretest (148.08 ms). The two-way interactions of time by group [*F*(1,46) = 0.79, *p* = 0.38, η_p_^2^ = 0.02], condition by group [*F*(1,46) = 2.07, *p* = 0.16, η_p_^2^ = 0.04], and the three-way interaction of group by time by condition [*F*(1,46) = 1.41, *p* = 0.24, η_p_^2^ = 0.03] were not significant.

For the Stroop interference effect of RTs, the main effect of time was significant, *F*(1,46) = 19.60, *p* < 0.001, η_p_^2^ = 0.30, indicating that the interference effect of RTs decreased in the posttest (107.30 ms) compared to the pretest (148.08 ms). The main effect of the group [*F*(1,46) = 2.07, *p* = 0.16, η_p_^2^ = 0.04] and interaction of time by group [*F*(1,46) = 1.41, *p* = 0.24, η_p_^2^ = 0.03] were not significant (Figure 3B).

### Event-related potential results

#### N450

**FIGURE 4:**
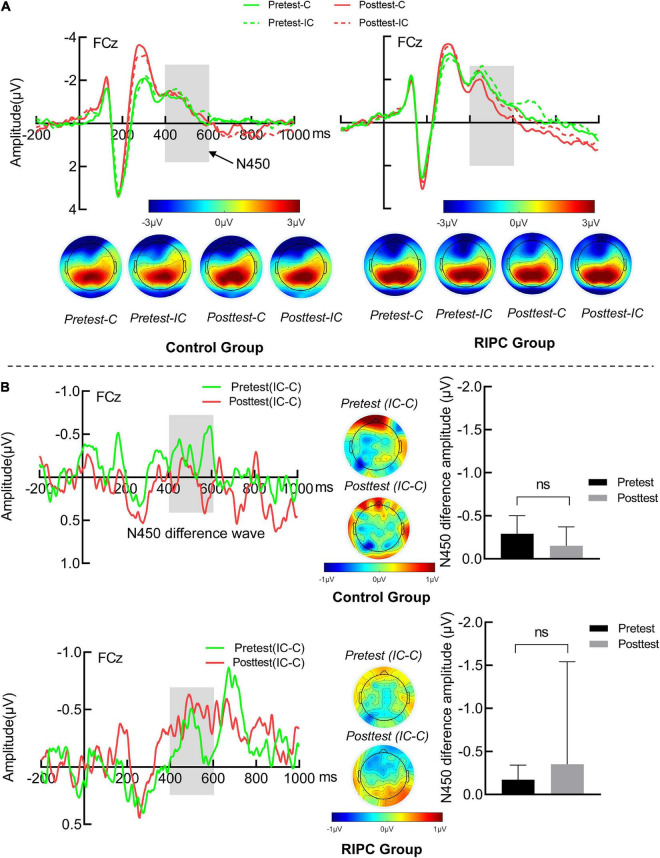
**(A)** The N450 grand average waveform (FCz) and topographical map of two groups. **(B)** The averaged difference wave and topographical map of N450 in two groups. The dark gray rectangles represent the measure time window of N450. Error bar represents the stand error; Pretest: before treatment, Posttest: immediately after treatment. C: Congruent, IC: Incongruent, IC-C: Incongruent minus Congruent. ns, no significance between the average difference wave of pretest and posttest.

For the N450, there was only a main effect of condition, *F*(1,46) = 4.17, *p* < 0.05, *η_p_^2^* = 0.08, indicating that N450 induced by incongruent trials (–0.68 μV) was greater than that by congruent trials (−0.44 μV), as shown in Figure 4A. Figure 4B shows other main effects and interactions were not significant, demonstrating that the N450 difference wave (incongruent minus congruent) was comparable between pretest and posttest in the control (–0.29 vs. –0.15 μV) and the RIPC (–0.17 vs. –0.34 μV) group. The main effect of time, *F*(1,46) = 0.64, *p* = 0.43, η_p_^2^ = 0.01; the two-way interactions of time by group [*F*(1,46) = 0.03, *p* = 0.86, η_p_^2^ = 0.001], condition by group [*F*(1,46) = 0.04, *p* = 0.85, η_p_^2^ = 0.001] and time by condition [*F*(1,46) = 0.02, *p* = 0.90, η_p_^2^ < 0.001], and the two-way interaction of group by time by condition [*F*(1,46) = 1.08, *p* = 0.30, η_p_^2^ = 0.02] were not significant.

#### Sustained potential

**FIGURE 5:**
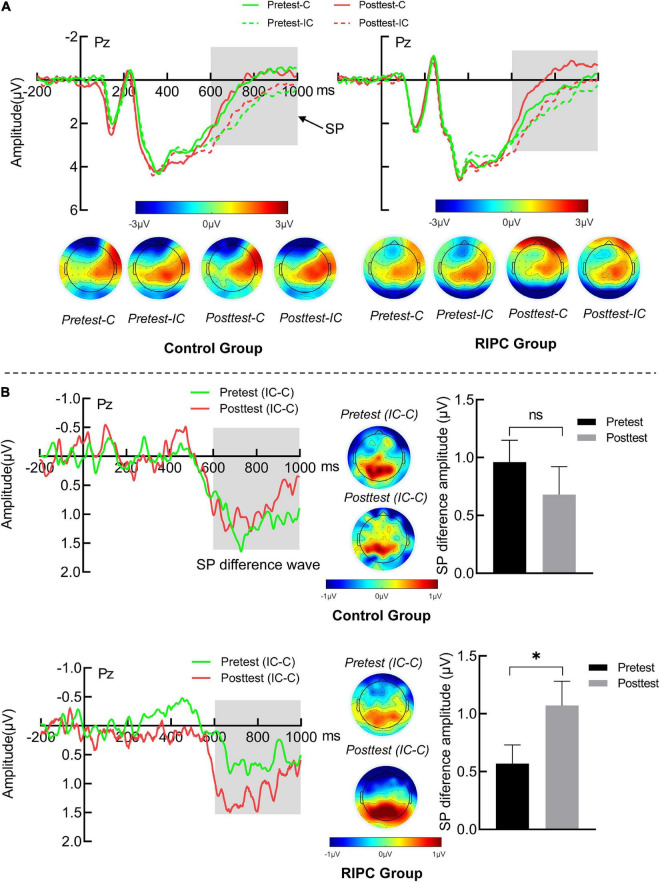
**(A)** The SP grand average waveform (Pz) and topographical map of two groups. **(B)** The averaged difference wave and topographical map of SP in two groups. The dark gray rectangles represent the measure time window of SP. Error bar represents the stand error; Pretest: before treatment, Posttest: immediately after treatment. C: Congruent, IC: Incongruent, IC-C: Incongruent minus Congruent. ns, no significance between the average difference wave of pretest and posttest. *Significant difference (*P* < 0.05) between the average difference wave of pretest and posttest.

For the SP, the main effect of the condition was found, *F*(1,46) = 52.18, *p* < 0.001, η_p_^2^ = 0.53, SP produced by incongruent trials (1.49 μV) was larger than congruent trials (0.67 μV), as shown in Figure 5A. Importantly, the three-way interaction of the group, time and the condition was significant, *F*(1,46) = 4.86, *p* = 0.03, η_p_^2^ = 0.10. Further simple effects analysis showed that, for the control group, the SP amplitude was larger for incongruent trials than for congruent trials in both pretest (1.93 vs. 0.97 μV, *p* < 0.001) and posttest (1.66 vs. 0.98 μV, *p* < 0.01). The amplitude of SP differential wave (incongruent minus congruent) was comparable between the pretest (0.96 μV) and posttest (0.68 μV). The RIPC group was the same as the control group, and the SP for incongruent trials was larger than that for congruent trials at both pretest (1.32 vs. 0.76 μV, *p* = 0.001) and posttest (1.03 vs. −0.04 μV, *p* < 0.001). However, the amplitude of the SP differential wave was enlarged at the posttest (1.06 μV) compared to the pretest (0.57 μV, *p* = 0.03), as shown in Figure 5B. The main effect of time, *F*(1,46) = 1.69, *p* = 0.20, η_p_^2^ = 0.04; the two-way interactions of time by group [*F*(1,46) = 0.66, *p* = 0.42, η_p_^2^ = 0.01], condition by group [*F*(1,46) = 0.001, *p* = 0.98, η_p_^2^ < 0.001] and time by condition [*F*(1,46) = 0.40, *p* = 0.53, η_p_^2^ < 0.01] were not significant.

### Correlation analysis

**FIGURE 6:**
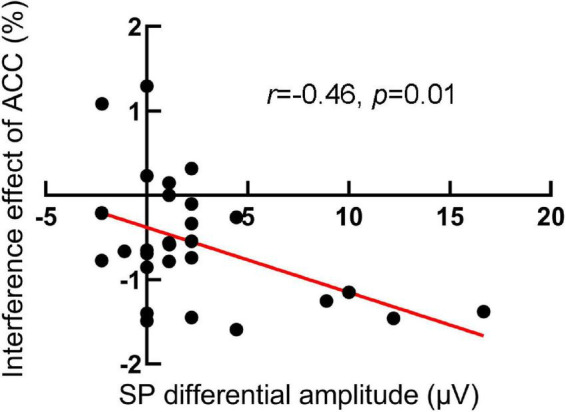
The scatter plot of the relationship between the interference effect of ACC and SP differential amplitude in the pretest for the RIPC group. The larger the differential SP amplitude, the smaller the interference effects of ACC.

We further compute the Pearson relation between the interference effects of RTs and ACC and SP differential amplitude (incongruent minus congruent), respectively, for the RIPC group. The results showed that in the pretest, the interference effects of ACC were negatively related to SP differential amplitude, *r* (28) = –0.46, *p* = 0.01, that is, the larger the differential SP amplitude, the smaller the interference effects of ACC (see Figure 6). Other relationships were not significant. In the pretest, RTs: *r* (28) = 0.19, *p* = 0.33; in the posttest, RTs: *r* (28) = –0.34, *p* = 0.08, ACC: *r* (28) = –0.27, *p* = 0.17.

## Discussion

Few studies suggested nearly no effect of RIPC on cognitive control of clinical patients or individuals in a special environment (e.g., Meybohm et al., 2013, 2018; Li et al., 2020). However, it remains uncertain whether healthy adults’ cognitive control could benefit from this treatment, although Sugimoto et al. (2021) proposed that the RIPC may be a potential treatment for improvement of this cognitive ability. Therefore, in the research, we seek empirical evidence of RIPC improving the cognitive control of healthy adults through the classic Stroop task and the ERP technique. To the best of our knowledge, this is the first research to evaluate the effect of the RIPC treatment on healthy individuals’ cognitive control using the ERP marker (perform 40 min per day for 7 days, 5 min 180 mm Hg ischemia, and 5 min reperfusion on a bilateral upper limb for four cycles). Behaviorally, participants’ RTs were longer and ACC was lower under the incongruent condition than under the congruent condition, indicating the phenomenon of the Stroop interference effect. In addition, compared with the pretest, the Stroop interference effect of RTs was reduced in the posttest. At the electrophysiological level, we mainly focused on the N450 and SP components, which indexed the conflict detection and resolution in the Stroop task respectively. Our ERP data showed that both N450 and SP elicited by incongruent trials were larger than those by congruent trials. Importantly, the SP differential amplitude between incongruent and congruent trials increased after participants received RIPC treatment compared with before RIPC, but this effect was not found in the control group.

The current behavioral data were consistent with previous studies of the Stroop effect (MacLeod, 1991; Hershman and Henik, 2019). When the ink color and meanings of words were incongruent, participants had to withhold accessing the meaning of words to overcome the conflict at the cost of slower response and reduced accuracy if they failed. Additionally, the response time was shortened and the interference effect from incongruent trials was reduced in the posttest compared with the pretest, which might be related to the extensive practice. Indeed, many previous Stroop studies also showed a reduction in the Stroop interference effect after participants extensively practice (MacLeod, 1991; Dulaney and Rogers, 1994; Davidson et al., 2003). However, we also should consider the effect of RIPC treatment on the interference effect reduction in the RIPC group. In this study, we included a control group that kept the same as the RIPC group in all aspects possible except for not receiving RIPC treatment. Thus, although it was difficult to solely distinguish the effect of practice and RIPC treatment on the Stroop interference reduction for the RIPC group. The difference in reduced interference effects between the two groups should be accounted for by the RIPC treatment because the practice effect actually would exist in the two groups in the meanwhile. This inference was indirectly supported by the results that, the magnitude of the reduced interference effect of RTs in the RIPC group (53.74 ms) was larger than that in the control group (27.81 ms), though this difference was not statistically significant.

Event-related potential data provide a window to observe the time course of the conflict processing in the Stroop task. Our electrophysiological data showed that the N450 amplitude of incongruent trials was larger than that of congruent trials, consistent with the previous work (West, 2003, 2004; Tillman and Wiens, 2011; Szûcs and Soltész, 2012). N450 was thought to be involved in the stimulus or response conflict monitoring processes (Lansbergen et al., 2007), so our N450 results suggested that participants perceived the conflict between the color and the meaning in incongruent trials. For the SP component, in line with the previous study (Liotti et al., 2000; Lansbergen et al., 2007), incongruent trials produced larger SP than congruent trials in the present study, representing the subsequent conflict solution stage or attentional control after conflict detection (West et al., 2005; Larson et al., 2009, 2014). More importantly, RIPC modulated the SP difference between congruent and incongruent trials, that is, the SP differential amplitude increased after RIPC treatment. But it is important to note that this result could not be interpreted by practice effects, as the same change did not occur in the control group. The enhanced SP difference has been associated with stronger conflict-resolving ability. For instance, individuals with schizophrenia had weaker cognitive control compared with healthy participants, which resulted in a smaller SP difference wave (McNeely et al., 2003). This viewpoint was also supported by the present study showing that increased SP differential amplitude between incongruent and congruent trials was associated with decreased interference effect of ACC in the pretest, indicating that the better conflict resolution, the less probability of responsive incorrect. In the posttest, although there was also a tendency for the interference effect of RTs and ACC to be negatively related to SP differential amplitude, but did not reach a significant level. This could be due to several factors, including the possibility that behavioral data reflect intermediate processes not necessarily reflected in the short-latency ERP component (Cacioppo and Tassinary, 1990). Taken together, combining the SP with the N450 results, we concluded that RIPC treatment might improve healthy adults’ ability to resolve but not monitor the conflict in the Stroop task.

Our data were also compatible with previous studies showing that RIPC treatment did not improve cognitive control at the behavioral level (Meybohm et al., 2013, 2018; Hudetz et al., 2015; Li et al., 2020; Sugimoto et al., 2021). For example, the executive function of attention in unacclimatized healthy adults both exposed to high altitudes and in the plain was not improved after accepted RIPC (Li et al., 2020). The behavioral performance of surgical patients treated with and without RIPC was comparable in the Stroop task (e.g., Hudetz et al., 2015; Meybohm et al., 2018). However, in our study, the positive effect of RIPC on cognitive control was confirmed by the electrophysiological marker (SP), suggesting that RIPC improved individuals’ conflict resolution ability. Therefore, the zero effect of RIPC on cognitive control in the present and previous studies might be due to behavioral indicators could not effectively capture the time course of conflict processing. The findings indicated that electrophysiological recordings were more sensitive to probing the subtle difference in the cognitive process than behavioral indicators.

Remote ischemic preconditioning is an inexpensive and non-invasive technique to alleviate brain and cognitive function impairment, operation of which is safe and simple, thus having good clinical application prospects (Moskowitz and Waeber, 2011). In addition to cognitive training methods such as game interventions, current stimulation, and music training, we offered PIRC as a potentially more promising option for individuals who required strong cognitive control or needed cognitive protection. Especially, some populations with emotional or behavioral disorders, such as cohorts with depression tendency and adolescents or elderly people who possess low cognitive control could use RIPC to improve cognitive control. Nonetheless, the physiological mechanism by which RIPC protects cognitive function had not been extensively recognized. For example, some authors hypothesized that biological factors such as bradykinin, adenosine, nitric oxide/calcitonin gene-related peptide, opioids, or endocannabinoids released by remote tissues can be carried via circulation to the target organ (Moskowitz and Waeber, 2011). Some authors also proposed that suppression of proinflammatory genes might be a candidate mechanism for the protective effect of RIPC on brain function (Konstantinov et al., 2004; He et al., 2017). Li et al. (2020) interpreted the effect of RIPC on alerting function of attention as improved brain microcirculation. The neural generator of SP was located in the frontal cortex (West, 2003; Lansbergen et al., 2007); thus, we speculated that the mechanism of facilitating the conflict resolution in the Stroop task may be the improved frontal microcirculation by RIPC treatment. Actually, this inference got supported to some extent by previous findings, for example, Ma et al. (2016) reported that RIPC treatment could protect ketamine-induced neuroapoptosis in the frontal cerebral cortex. A recent study by Oh et al. (2017) revealed that RIPC could increase cerebral oxygenation of the frontal lobe. However, the mechanism of this cognitive promotion should be further defined accurately in the future, by collecting and analyzing more physiological indicators such as blood and neurotransmitter before and after RIPC treatment.

Other limitations of this study should also be addressed. First, at the behavioral level, the effects of practice and RIPC treatment on the Stroop interference effect of RTs were mixed, which made it difficult to quantify the real effect of RIPC treatment on cognitive control. In the future, this problem can be avoided to some extent by using different tasks but involving in the same cognitive process of conflict processing, such as the Stroop and flanker task in pretest and posttest respectively (Eriksen and Eriksen, 1974; Tillman and Wiens, 2011), or decrease the presentation probability of incongruent trials to weak the practice effects. Second, the sample size was small, and all participants were non-clinical individuals. It remained unclear whether the results could be used to guide the clinic therapy. Thus, it was necessary to recruit clinical populations with impaired cognitive control such as individuals with attention deficit hyperactivity disorder (ADHD) and autism to extend our conclusion. Third, the participants were all young men, so the effects of gender and age should be considered. Finally, further study was necessary to explore a possibly better RIPC treatment program to protect cognitive control, given the effect of RIPC was not reflected in behavioral performance.

## Conclusion

To sum up, in this study, we examined the effect of RIPC treatment on healthy adults’ cognitive control. We conclude that healthy adults’ cognitive control especially the conflict resolving in the Stroop task could be improved by RIPC treatment, which is reflected in the increase of SP component at the electrophysiological level. In the future, the RIPC could be an alternative treatment for improving healthy adults’ cognitive control, meanwhile, the researcher also should further investigate the exact physiological mechanism of RIPC’s protective effect on cognitive control, and other populations, such as individuals with ADHD and autism, should be included.

## Data availability statement

The raw data supporting the conclusions of this article will be made available by the authors, without undue reservation.

## Ethics statement

The studies involving human participants were reviewed and approved by the Medical Ethics Committee of Army Medical University. The patients/participants provided their written informed consent to participate in this study.

## Author contributions

PL and YW conceived the idea for the study, supervised the RIPC treatment, and approved the version to be published. YL designed the experiment and wrote the manuscript. JH, PH, and ZZ revised the manuscript. YL, YW, JH, PH, SZ, HD, ZZ, and JX performed and obtained the data. YL and JH analyzed the data and drew the figures. All authors have approved its submission and publication.

## Footnotes

1
http://www.biosemi.com/headcap.htm
